# Neuroprotective effects of a lead compound from coral via modulation of the orphan nuclear receptor Nurr1

**DOI:** 10.1111/cns.14025

**Published:** 2022-11-23

**Authors:** Jian‐Wei Su, Pei Yang, Mei‐Mei Xing, Bao Chen, Xia‐Hong Xie, Jian‐Hua Ding, Ming Lu, Yang Liu, Yue‐Wei Guo, Gang Hu

**Affiliations:** ^1^ Department of Pharmacology School of Medicine and Holistic Integrative Medicine, Nanjing University of Chinese Medicine Nanjing Jiangsu China; ^2^ State Key Laboratory of Drug Research Shanghai Institute of Materia Medica, Chinese Academy of Sciences Shanghai China; ^3^ Jiangsu Key Laboratory of Neurodegeneration, Department of Pharmacology Nanjing Medical University Nanjing China

**Keywords:** *C. elegans*, corals‐derived compounds, neuroprotection, Nurr1, SH‐SY5Y cells

## Abstract

**Aims:**

To screen coral‐derived compounds with neuroprotective activity and clarify the potential mechanism of lead compounds.

**Methods:**

The lead compounds with neuroprotective effects were screened by H_2_O_2_ and 1‐methyl‐4‐phenyl‐1,2,3,6‐tetrahydropyridine (MPP^+^)‐induced cell damage models in SH‐SY5Y cells. CCK8 and LDH assays were used to detect cell viability. The anti‐apoptosis of lead compounds was evaluated by flow cytometry. JC‐1 and MitoSox assays were performed to examine the changes in mitochondrial membrane potential and mitochondrial ROS level. Survival of primary cortical and dopaminergic midbrain neurons was measured by MAP2 and TH immunoreactivities. The *Caenorhabditis elegans* (*C. elegans*) model was established to determine the effect of lead compounds on dopaminergic neurons and behavior changes.

**Results:**

Three compounds (No. 63, 68, and 74), derived from marine corals, could markedly alleviate the cell damage and notably reverse the loss of worm dopaminergic neurons. Further investigation indicated that compound 63 could promote the expression of Nurr1 and inhibit neuronal apoptosis signaling pathways.

**Conclusion:**

Lead compounds from marine corals exerted significant neuroprotective effects， which indicated that coral might be a new and potential resource for screening and isolating novel natural compounds with neuroprotective effects. Furthermore, this study also provided a new strategy for the clinical treatment of neurodegenerative diseases such as Parkinson's disease.

## Graphical abstract

Compound 63, a novel natural compound with excellent neuroprotection ability, was screened from marine coral. It could markedly upregulate the expression of Nurr1 and inhibit neuronal apoptosis signaling pathways, which provided a novel strategy for drug discovery in treating Parkinson's disease.

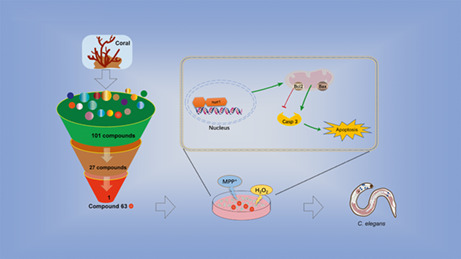

## 1 INTRODUCTION

Neurodegenerative diseases are a group of devastating neurological disorders characterized by the progressive loss of neuronal structure or function.^1^ Although the exact etiology of neurodegenerative diseases is little known, recent evidence has shown that oxidative stress notably accelerates its development.^2^, ^3^ Oxidative stress, a condition produced by the imbalance between oxidants and antioxidants in a biological system, occurs as a result of the excess level of reactive species (ROS) or improper function of the antioxidant system.^4^ The high inspired oxygen, leading to mitochondrial dysfunction, is responsible for devastating neurodegenerative disease progress, such as Parkinson's disease alpha‐synucleinopathy.^5^ Parkinson's disease (PD), the second most common neurodegenerative disease, seriously affects >1% of the population over 65 years old.^6^ Currently, the available treatments for PD have provided only symptomatic relief and cannot slow down the disease progression. Hence, it is essential to develop new effective drugs for the treatment of neurodegenerative diseases.

Marine drug resources have promising development prospects and potential applications due to their novel chemical structures and unique biological activities.^7^, ^8^ Recently, a large number of marine natural products have been proved to have a variety of therapeutical properties in PD treatment.^9^, ^10^, ^11^, ^12^ Coral reefs are the largest ecosystems in the oceans and their future is of great concern. Soft corals have displayed a vital role in the innovation of lead compounds for drug development, which rely on their diverse defense mechanisms^13^: first, they can defend competitors from taking over their living space; second, they have the potential to restrain the pathogenic and parasitic microorganisms by synthesizing and accumulating toxic secondary metabolites in the body; moreover, they can maintain their survival through secreting the metabolites into the surrounding environment.^14^ Currently, soft coral‐derived 11‐dehydrosphingosine can protect 6‐hydroxydopamine (6‐OHDA)‐mediated neuronal damage by upregulating the expression of PI3‐K/Akt and DJ‐1 proteins, which suggests its neuroprotective effect in PD models.^15^, ^16^ Likewise, sterols from soft corals have revealed interesting biological activities including anti‐inflammation, anti‐tuberculosis, anti‐diabetic, and anti‐cancer.^17^, ^18^, ^19^ Accordingly, the remarkable diversity of bioactive compounds, isolated from marine soft corals, will offer a new perspective on the future direction of drug discovery.

In this study, 101 compounds from marine corals were screened for neuroprotective biological activities. Furthermore, databases predicted that nuclear receptor subfamily 4 group A member 2 (Nurr1 or Nr4A2) might be the potential target of lead compounds.^20^, ^21^, ^22^ These findings would be helpful to screen novel lead compounds with neuroprotective effects from coral and provide valuable insight for the treatment of neurodegenerative diseases such as PD.

## 2 MATERIALS AND METHODS

### 2.1 Animals

Wild‐type N2, transgenic BZ555 *C. elegans* (P[dat‐1]::GFP, bright green fluorescent protein expression in DA neuronal soma and processes), and OP50 strains were obtained from Nanjing University of Chinese Medicine. *C. elegans* were maintained on nematode growth media (NGM) and fed with *Escherichia coli* OP50 strain as a food source at 20°C. The synchronized population of animals was isolated from adult worms by bleaching solution (10% NaClO and 10% NaOH) for 10 min and washed with M9 buffer three times, incubated at 20°C overnight to obtain newly hatched L1 larva.

### 2.2 Reagents

All nature compounds derived from marine corals were provided by the Shanghai Institute of Materia Medica, Chinese Academy of Sciences. 1‐Methyl‐4‐phenyl‐1,2,3,6‐tetrahydropyridine (MPP^+^) was purchased from GLPBIO (GC18188). Dulbecco's modified Eagle's medium (DMEM) and fetal bovine serum (FBS) were bought from Gibco (C11995500BT, 10099–141). Nematode growth media (NGM) contained 50.0 mM NaCl, 1.0 mM CaCl_2_, 1.0 mM MgSO_4_, 19.9 mM KH_2_PO_4_, 5.1 mM K_2_HPO_4_, 2.5 g/L peptones, and 5 mg/L cholesterol, pH 6.0. A lactate dehydrogenase (LDH) assay kit was provided by Nanjing Jiancheng Bioengineering Institute (A020‐1‐2). Annexin V‐FITC/PI Apoptosis Detection Kit, HiScript III‐RT SuperMix for qPCR (+gDNA wiper), and ChamQ SYBR qPCR Master Mix (High ROX Premixed) were obtained from Vazyme (A211‐02, R323‐01, Q341‐02). MAP2, MitoSox, and JC‐1 fluorescent probes were purchased from Invitrogen (USA, SC‐32791, M36008, T3168). SOD and GSH/GSSG kits were bought from Beyotime Biotechnology (Shanghai, China, S0087, S0053).

### 2.3 SH‐SY5Y cell and primary neuron culture

SH‐SY5Y cells were cultured with DMEM medium (containing 10% FBS and 1% penicillin–streptomycin) at 37°C in an incubator with 5% CO_2_. Primary neuron cultures were prepared as follows. Briefly, whole brains were extracted from C57/BL6 mouse embryos at embryonic day 14 (E14). The cortical and dopaminergic midbrain neurons were planted from the brain by gently removing the meninges in a cold DMEM medium, shredding, and treating with 0.25% trypsin for 20 min at 37°C in an aseptic condition. The cells were suspended in supplemented neurobasal medium and cultured in the cell incubator.

### 2.4 Cell viability assay

The cell counting Kit‐8 (CCK‐8) assay (Selleck, Houston, TX, USA, B34034) was performed to assess cell proliferation according to the manufacturer's instructions. SH‐SY5Y cells were seeded in 96‐well plates (NEST Biotechnology Co. Ltd., Wuxi, China) at a density of 5 × 10^3^ cells per well in 100 μl of DMEM medium for 24 h and treated with different compounds at a concentration of 1 μM. After 24 h, 10 μl of CCK‐8 reagent was added to each well and then cultured for 2 h at 37°C in the dark. The absorbance was analyzed at 450 nm using Varioskan Flash (Thermo). Three independent experiments were performed per condition.

### 2.5 Lactate dehydrogenase (LDH) assay

Lactate dehydrogenase release, as an indicator of cell membrane permeability, was measured with the LDH assay kit. Primary neurons or SH‐SY5Y cells, cultured in a 96‐well plate following the treatment with compounds for 24 h, were transferred to a new 96‐well plate and incubated with LDH reagent for 30 min according to the manufacturer's instructions. Results were measured at 490 nm using a Varioskan Flash.

### 2.6 Flow cytometry

Annexin V‐FITC/PI Apoptosis Detection Kit was used to detect cell apoptosis by flow cytometry using a Guava Easy Cyte 6‐2L (Merck). The cells were collected, washed with cold PBS, and suspended with 100 μl 1 × Binding Buffer. Following this, the cells were stained with 5 μl of Annexin‐V‐FITC and PI for 10 min at room temperature in the dark. The stained cells were analyzed by flow cytometry acquiring gated 5000 events. Gated cells were separated into four quadrants: early apoptotic cells (Annexin‐V positive/PI negative), necrotic cells (Annexin‐V negative/PI positive), late apoptotic cells (Annexin‐V positive/PI positive), and viable cells (Annexin‐V negative/PI negative).

### 2.7 Western blot

Total protein was extracted by RIPA protein lysis buffer (R0020, Solarbio Science & Technology Co. Ltd., Beijing, China) and quantified by the BCA methods. Protein bands were separated by SDS‐PAGE and then transferred to a polyvinylidene difluoride (PVDF) membrane. After blocking with 5% bovine serum albumin (BSA) for 1 h, the PVDF membrane was incubated with primary antibodies at 4°C overnight. After being washed three times in TBST, the membrane was incubated with secondary antibodies for 2 h at room temperature. Finally, the bands were visualized with enhanced chemiluminescence (ECL) Kit using an Image Quant LAS 4000 mini (GE). The intensity of western bands was quantified by ImageJ 5.1 software.

### 2.8 Cell immunofluorescence

Cells were washed with PBS buffer twice and fixed with 4% paraformaldehyde for 30 min at room temperature. Each slide was blocked with 5% BSA in PBST (containing 0.3% Triton 100) solution for 1 h and incubated with primary antibody at 4°C overnight. Then, cells were washed and incubated with fluorescent secondary antibody (1:1000) at room temperature for 2 h in the dark. The stained cells were observed by a stereomicroscope (Olympus BX51). Primary antibodies used in this experiment were as follows: Hoechst (1:1000, Sigma, 14533), JC‐1 (1: 2000, Invitrogen, 65‐0851‐38), MitoSox (1: 1000, Invitrogen, M36008), mouse anti‐TH (1: 1000, Sigma, T1299), mouse anti‐MAP2 (1: 200, Proteintech, 17490‐1‐AP). The fluorescent secondary antibodies such as red fluorescent mouse secondary antibody Alexa Fluor555and green fluorescent rabbit secondary antibody Alexa Fluor 488 were applied to this experiment. TH‐positive neurons and neurons' neurite length were calculated by Image‐Pro Plus 6.0 software.

### 2.9 RT‐qPCR

Total cell RNA in each group was extracted with Trizol reagent (YFX, YFXM0011) and the RNA concentration was measured using a spectrophotometer (Nano Vue). Subsequently, the total RNA was reverse‐transcribed into cDNA by HiScript III‐RT SuperMix for qPCR (+gDNA wiper) according to the manufacturer's instructions. GAPDH and β‐actin were used as the housekeeping genes. The primer sequences used for the RT‐qPCR are shown in Table S1.

### 2.10 Lifespan assay

Synchronized L2 larvae of N2 and BZ555 type *C. elegans* were transferred to NGM plates containing 8 mM MPP^+^ with the presence of compounds 63, 68, and 74. The surviving worms were counted after 48 h treatment. The numbers of live and dead worms were counted and recorded daily. And the lifespan was calculated until all worms died.

### 2.11 Behavioral assessment

Well‐fed worms with intact DA neural circuitry moved slower in the presence of bacterial food than in its absence. This basal slowing response was recorded as described previously.^23^ N2 worms were treated with compounds 63, 68, or 74 (5 μM) for 48 h, the worms were washed 3 times with M9 buffer to remove excess OP50 bacteria. The basal slowing rate of *C. elegans* assay was carried in the NGM plate with or without bacteria. The worms were transferred to the plates and stood for 5 min to recover. Then, the body bending times of all nematodes within 10 s were recorded, and nematodes with or without bacteria in each group were calculated. Swimming induced paralysis (SWIP) test was carried out as follows. After being treated with 8 mM MPP^+^ and compounds 63, 68, or 74 (5 μM) for 48 h, *C. elegans* were firstly immersed into a drop of 200 mM sucrose solution on a plate with platinum wire. Subsequently, the time was calculated when worms began to immerse in the sucrose solution. The numbers of nematodes with SWIP were recorded every 2 min. Statistical criteria were that the nematodes stopped moving and sank to the bottom of the solution.

### 2.12 GSH, GSH/GSSG ratio, and SOD analysis

SH‐SY5Y cells were harvested, washed with 0.1 M PBS, and then centrifuged at 10,000 ***g*** for 10 min. The supernatant was discarded and cell precipitation was ultrasonically broken to obtain the homogenate at 4°C. The concentrations of superoxide dismutase (SOD), total glutathione (T‐GSH), reduced glutathione (GSH), and oxidized disulfide (GSSG) were measured according to the commercial assay kit procedures.

### 2.13 Cell transfection

Before transfection, SH‐SY5Y cells were transferred into Opti‐MEM® (Thermo Fisher Scientific Inc.) for 2 h in the DMEM medium. To prepare transfection complexes, 2 μl siRNA‐Mate transfection reagent (GenePharma, Co., Ltd.) and 40 pmol siRNA of Nurr1 or negative control (NC) were separately diluted, gently mixed, and incubated for 15–20 min, according to the instructions provided by the manufacturer. The cells were harvested after 24 h culture.

### 2.14 Statistical analysis

Data were analyzed using GraphPad Prism 9.0 software and showed as mean ± SEM. Fluorescence and western blot images were analyzed by ImageJ 5.1 software. The survival rate curves were analyzed with a log‐rank test and the differences between different groups were analyzed by the one‐way anova test. *p* < 0.05 was considered to be statistically significant.

## 3 RESULTS

### 3.1 Neuroprotective activity of 101 compounds from marine coral

**FIGURE 1:**
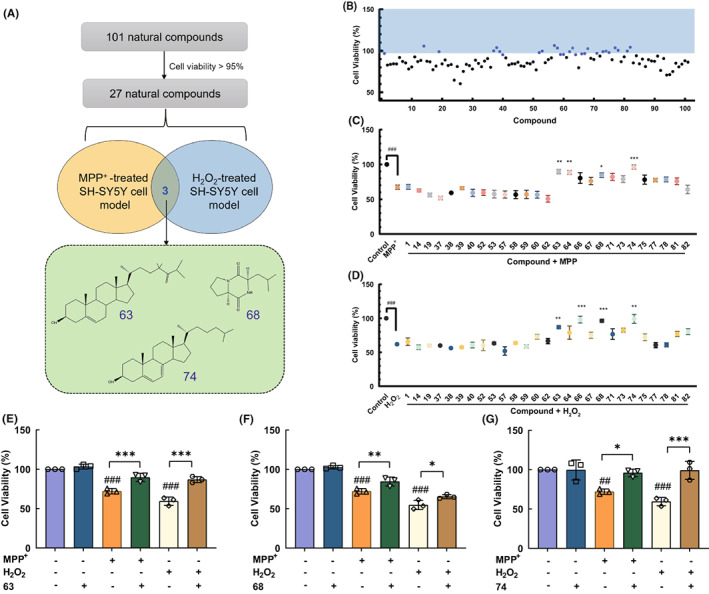
Neuroprotective activity of novel natural compounds from marine coral. (A) Schematic diagram of natural compounds screening process. (B) The cell viability of SH‐SY5Y cells after the treatment of 101 natural compounds. (C, D) The cell viability of SH‐SY5Y cells treated with compounds from the primary screening and MPP^+^ (500 μM) or H_2_O_2_ (100 μM). (E–G) The cell viability of SH‐SY5Y cells treated with compounds 63, 68, 74, and MPP^+^ or H_2_O_2_. Differences between the treatment groups were assessed using one‐way anovas, followed by Dunnett's multiple comparisons test or Šídák's multiple comparisons test. Data were expressed as mean ± SEM, ###*p* < 0.001 compared with the control group; *p < 0.05, **p < 0.01, and ***p < 0.001 compared with the MPP^+^/H_2_O_2_ group. All experiments were performed in triplicate.

1‐Methyl‐4‐phenylpyridinium (MPP^+^), an exogenous neurotoxin, has been widely used to evaluate the neuroprotective activity by inactivating the enzyme complex I of the respiratory chain in mitochondria.^24^, ^25^ One hundred and one natural compounds derived from marine corals were incubated with SH‐SY5Y cells at a concentration of 1 μM for 24 h, and the cell viability of each group was detected by the CCK‐8 method (Figure 1A,B, Table S2). SH‐SY5Y cell damage models were successfully established by 500 μM MPP^+^ or 100 μM H_2_O_2_ (Figure S1A,B). Subsequently, the cell viability of compounds >95% was further exposed to MPP^+^ or H_2_O_2_ for 24 h (Figure 1C,D **)**. In the MPP^+^‐induced SH‐SY5Y cell model, the pre‐treatment of compounds 63, 68, and 74 markedly enhanced the viability of SH‐SY5Y cells to 89.73% (*p* < 0.001), 84.57% (*p* < 0.01), 95.90% (*p* < 0.05), respectively. Besides, compared with H_2_O_2_‐treated SH‐SY5Y cells, the cell viability of compounds 63, 68, and 74 groups notably increased to 86.93% (*p* < 0.001), 65.43% (*p* < 0.05), 99.11% (*p* < 0.001), respectively (Figure 1E–G). The results indicated that compounds 63, 68, and 74 could significantly reduce the MPP^+^ and H_2_O_2_‐induced SH‐SY5Y cell damage.

### 3.2 Improvement of compounds 63, 68, and 74 on the MPP^+^‐induced cell mitochondrial dysfunction

To verify the neuroprotective effect of three compounds, SH‐SY5Y cells were pre‐treated with different concentrations of compounds (1 nM, 10 nM, 100 nM, 1 μM, 5 μM) for 1 h and co‐incubated with MPP^+^ for 24 h. The results showed that compounds 63, 68, and 74 significantly inhibited the MPP^+^‐induced cell damage (Figure S2A–D). The next cell experiments were performed at 1 μM of all compounds, at which concentration the MPP^+^/H_2_O_2_‐induced cell damage could be significantly improved. During the process of cell apoptosis or death, the cell membrane will be damaged, resulting in the release of enzymes in the cytoplasm and the increase of lactate dehydrogenase (LDH). The supernatant of SH‐SY5Y cells was collected to detect the LDH release. The results indicated that compounds 63, 68, and 74 remarkably reduced the level of LDH in MPP^+^‐induced SH‐SY5Y cells (Figure S2D), which suggested that they could decrease the MPP^+^‐induced cytotoxicity on SH‐SY5Y cells.

**FIGURE 2:**
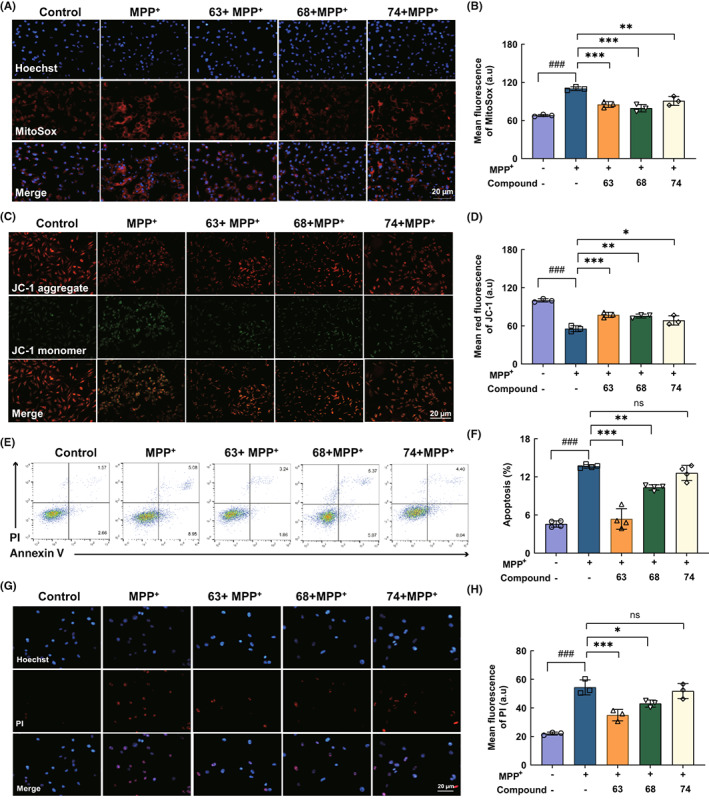
Alleviation of compounds 63, 68, and 74 on mitochondrial dysfunction and apoptosis in MPP^+^‐induced SH‐SY5Y cells. (A) Representative immunofluorescence images of MitoSox (red) and Hoechst (blue) in SH‐SY5Y cells. (B) Mean fluorescence intensity analysis of mitoSox. (C, D) Representative images of JC‐1 (JC‐1 aggregate, red; JC‐1 monomer, green) and mean red fluorescence intensity analysis of JC‐1 (D). (E, F) Flow cytometric analyses of PI‐Annexin V staining of apoptotic SH‐SY5Y cells. Cells for Annexin V^+^/PI^−^ and Annexin V^+^/PI^−^ were both considered to be apoptotic. (G, H) Representative immunofluorescence images of PI (G) and Mean fluorescence intensity analysis (H). Scale bar = 20 μm. Differences between the treatment groups were assessed using one‐way anovas, followed by Dunnett's multiple comparisons test. Data were expressed as mean ± SEM, ^###^ *p* < 0.001 compared with the control group; *p < 0.05, **p < 0.01, and ***p < 0.001 compared with the MPP^+^ group. All experiments were performed in triplicate.

Mitochondrial dysfunction is a causative factor of neurodegenerative diseases. To explore the effect of three compounds on mitochondrial function, cells were pre‐treated with 1 μM of compounds 63, 68, or 74 for 1 h and exposed to 500 μM MPP^+^ for 24 h. The mitochondrial ROS levels were detected using the fluorescent probe MitoSox Red (Figure 2A). And the results showed that ROS mean fluorescence intensity (MFI) in the MPP^+^ group was increased to 110.15 a.u. After the treatment of compounds 63, 68, and 74, ROS MFIs were recovered to 85.07, 79.45, and 90.78 a.u., respectively (Figure 2B). Additionally, mitochondrial membrane potential (Δψm) was determined by JC‐1 staining (Figure 2C). Generally, JC‐1 aggregates to polymers in the mitochondria and emits strong red fluorescence in the healthy cells. While the cells are damaged, the mitochondrial membrane potential is downregulated and JC‐1 can exist in the form of monomers that emits green fluorescence. In the MPP^+^‐induced SH‐SY5Y cell model, the JC‐1 red fluorescence intensity declined to 55.48% and green fluorescence intensity was increased. After treated with compounds 63, 68, and 74, the red MFIs of JC‐1 were markedly recovered to 77.23% (*p* < 0.001), 75.46% (*p* < 0.01), and 68.61% (*p* < 0.05), respectively (Figure 2D). The compounds 63, 68, and 74 could markedly alleviate the decrease of mitochondrial membrane potential caused by MPP^+^ and significantly reduce cellular oxidative damage. MitoSox and JC‐1 fluorescence results showed that the three compounds could notably alleviate the oxidative damage of SH‐SY5Y cells.

The apoptosis of SH‐SY5Y cells was detected by AV‐PI staining. The results showed that the apoptosis rate of SH‐SY5Y cells induced by MPP^+^ was 13.67%. After treated with compounds 63, 68, and 74, the apoptosis rates significantly decreased to 5.37% (*p* < 0.001), 10.34% (*p* < 0.01), and 12.63% (*p* > 0.5), respectively (Figure 2E,F). The results of PI fluorescence staining were consistent with those of flow cytometry (Figure 2G,H), which further verified the neuroprotective effect of compounds 63 and 68 on SH‐SY5Y cells.

### 3.3 Neuroprotective effects of compounds 63, 68, and 74 on primary neurons

**FIGURE 3:**
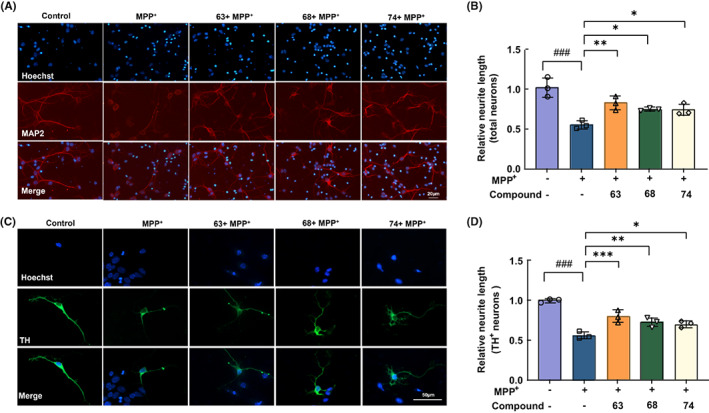
Neuroprotective effects of compounds 63, 68, and 74 on primary neurons. (A, B) Representative immunofluorescence images of MAP2 (A) and mean fluorescence intensity analysis (B). Scale bar = 20 μm. (C, D) Representative immunofluorescence images of TH and mean fluorescence intensity analysis. Scale bar = 50 μm. Nuclei were stained with Hoechst (blue). Differences between the treatment groups were assessed using one‐way anovas, followed by Dunnett's multiple comparisons test. Data were expressed as mean ± SEM, ^###^ *p* < 0.001 compared with the control group; *p < 0.05, **p < 0.01, and ***p < 0.001 compared with the MPP^+^ group. All experiments were performed in triplicate.

To explore the protective effect of compounds 63, 68, and 74 on neurons, primary neurons were extracted from wild‐type C57/BL6 pregnant mice and co‐cultured with three compounds. The CCK‐8 results indicated that compounds 63 and 68 could notably reduce the MPP^+^‐caused primary neuron damage at a concentration of 1 μM while compound 74 at a concentration of 100 nM (Figure S2E–G). LDH release results indicated that compounds 63, 68, and 74 markedly alleviated the MPP^+^‐induced neuronal cytotoxicity (Figure S2H). Next, neuronal synaptic strength was detected by microtubule‐associated protein 2 (MAP2) and tyrosine hydroxylase (TH) immuno‐fluorescence in primary cortical and dopaminergic midbrain neurons (Figure 3A,C). The length of neuronal synapses was decreased to 55.15% when exposed to MPP^+^, while compounds 63, 68, and 74 could significantly increase total neuron length to 86.23% (*p* < 0.01), 74.54% (*p* < 0.05) and 74.18% (*p* < 0.05), respectively (Figure 3B). Moreover, TH‐positive neurons in the midbrain remarkably recovered to 80.31% (*p* < 0.001), 72.79% (*p* < 0.01), and 70.46% (*p* < 0.05) in compounds 63, 68, and 74 treated groups, respectively (Figure 3D). Collectively, these findings suggested that compounds 63, 68, and 74 exerted excellent neuroprotective effects on neurons.

### 3.4 Alleviation of compounds 63 and 74 on the loss of worm dopaminergic neurons challenged by MPP^+^ in the *C. elegans* model

**FIGURE 4:**
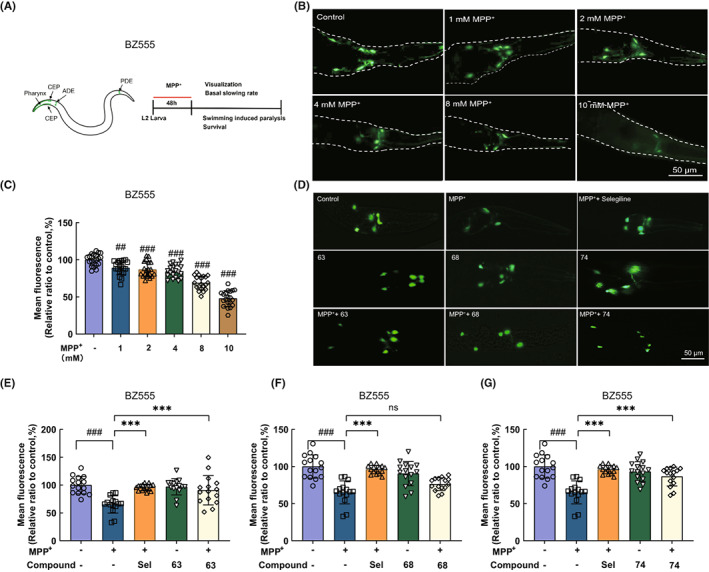
Compounds 63 and 74 reverse the loss of MPP^+^‐treated dopaminergic neurons in the *Caenorhabditis elegans* model. (A) Illustrations of the DA neuron anatomy in *C. elegans*. (B) Representative fluorescence images of different concentrations of MPP^+^ on dopamine neurons of BZ555 *C. elegans* (dat‐1p::GFP). (C) Statistics of mean fluorescence intensity of dopamine neurons in BZ555 *C. elegans* model. (D) GFP expression patterns of MPP^+^ (8 mM)‐treated transgenic strain BZ555, and treated with positive control medicine (selegiline, 5 μM), compounds 63 (5 μM), 68 (5 μM), or 74 (5 μM). Scale bar = 50 μm. (E–G) Mean fluorescence intensity analysis of DA neurons in BZ555 *C. elegans* model. Differences between the treatment groups were assessed using one‐way anovas, followed by Dunnett's multiple comparisons test or Šídák's multiple comparisons test. Data were expressed as mean ± SEM, ^###^ *p* < 0.001 compared with the control group; ***p < 0.001 compared with the MPP^+^ group. The experiment was performed independently at least three times (The number of worms = 15–20 animals/group per replicate).

In this study, BZ555 *C. elegans* (P[dat‐1]::GFP), in which dopaminergic neurons were labeled by GFP, was used to examine the dopaminergic neuroprotection effects of three compounds as described previously.^26^ MPP^+^ selectively induced the degeneration of dopaminergic neurons (Figure 4A,B). The exposure of MPP^+^ dose‐dependently (1–10 mM) increased the neurodegeneration in the L2 larvae of BZ555 (Figure 4B). The dopaminergic neuron damage model of *C. elegans* was successfully established after the treatment with 8 mM of MPP^+^, in which fluorescence intensity was reduced to 68.84% (Figure 4C). Compared with the MPP^+^‐induced group, higher GFP intensities were detected after the treatment of compounds 63, 68, and 74 (5 μM) (Figure 4D). And the mean fluorescence intensity of the worms treated with compounds 63, 68, and 74 significantly increased to 90.87% (*p* < 0.001), 76.35% (*p* > 0.05), and 86.51% (*p* < 0.001), respectively (Figure 4E–G). The results indicated that compounds 63 and 74 exhibited remarkable neuroprotection on MPP^+^‐induced dopaminergic neurodegeneration.

### 3.5 Amelioration of compounds 63 and 68 on the MPP^+^‐induced behavioral disorder of *C. elegans*

**FIGURE 5:**
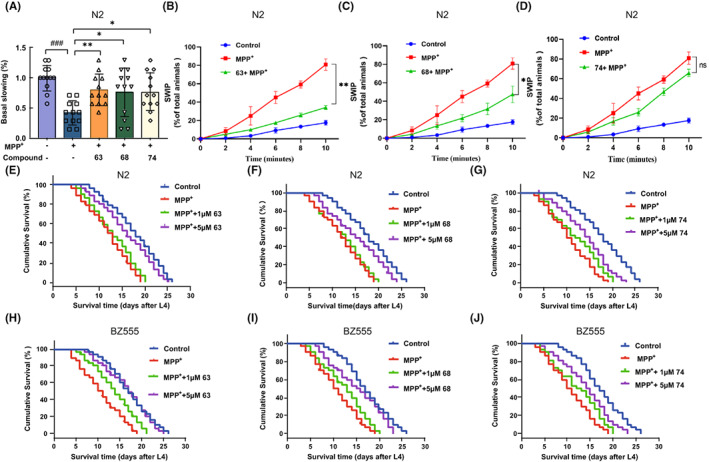
Compounds 63 and 68 improve behavioral disorder and MPP^+^‐induced lifespan reduction in the *Caenorhabditis elegans* model. (A) The basal slowing rate in MPP^+^‐induced N2 *C. elegans* and treated with 63, 68, and 74. (B–D) The percent of N2 *C.elegans* exhibiting SWIP. (E–J) Survival curves in MPP^+^‐induced N2 and BZ555 *C. elegans* and treated with compounds 63, 68, and 74. Differences between the treatment groups were assessed using one‐way anovas, followed by Dunnett's multiple comparisons test. Data were expressed as mean ± SEM, ^###^ *p* < 0.001 compared with the control group; *p < 0.05, **p < 0.01 compared with the MPP^+^ group. The experiment was performed independently at least three times (The number of worms = 12–20 animals/group per replicate).

MPP^+^ could cause the selective degeneration of dopaminergic neurons, while worms lacking dopamine displayed related behavioral disorders.^27^, ^28^ Thus, dopamine‐dependent behaviors including the basal slowing response and swimming‐induced paralysis were further examined. Similar to previous studies, MPP^+^ exposure significantly decreased the basal slowing rate compared with normal worms (*p* < 0.001) (Figure 5A). Worms coincubating with MPP^+^ and compounds 63 or 68 showed a significant recovery in the basal slowing rate and swimming‐induced paralysis compared with only MPP^+^‐induced worms (*p* < 0.05). In contrast, worms treated with 5 μM compound 74 slightly improved swimming induced paralysis with no significant difference (*p* > 0.05) (Figure 5B–D). These results indicated that compounds 63 and 68 could rescue the degenerated dopaminergic neuron and alleviate the MPP^+^‐induced dopaminergic neuron injury.

### 3.6 Rescue of compounds 63 and 68 on lifespan challenged by MPP^+^ in the *C. elegans* model

Previous studies have proved that MPP^+^ could induce dopaminergic neuron death and worm lethality.^29^, ^30^ The mean lifespans of N2 and BZ555 worms were approximately 20.8 and 19.2 days under normal conditions, respectively. After the exposure to 8 mM MPP^+^, survival rates of N2 and BZ555 worms were significantly shortened by 7.9 days (*p* < 0.01) and 6.3 days (*p* < 0.05), respectively. However, with the treatment of compounds 63 and 68, the worm lifespans of N2 and BZ555 nematodes were markedly increased by 6.9 days (*p* < 0.01) and 5.4 days (*p* < 0.05), respectively (Figure 5E,F,H,I). And there was no significant difference in the compound 74 treated group (Figure 5G,J).

### 3.7 Inhibition of compound 63 on MPP^+^‐induced apoptosis by Nurr1

Compounds 63 and 74 were identified as sterols and compound 68 was an alkaloid (Structures are shown in Figure 1A). Previous studies have shown that sterol compounds play an important role involved in the process of neuroprotection, such as cognition, memory, and neurogenesis.^31^, ^32^, ^33^ Moreover, they are reported to be tightly associated with a reduced risk of Parkinson's disease and Alzheimer's disease.^34^, ^35^ Compared with compounds 68 and 74, compound 63 performed a better effect on reversing MPP^+^‐induced mitochondrial oxidative damage (Figure 2A–D) and apoptosis (Figure 2E–H). In vivo studies of *C. elegans* also showed that compound 63 could significantly improve the damage of MPP^+^‐induced nematode dopamine neurons, prolong the survival rate, and ameliorate the behavioral disorder of *C. elegans* (Figures 4 and 5). Collectively, compound 63 was selected as the lead compound for further study.

**FIGURE 6:**
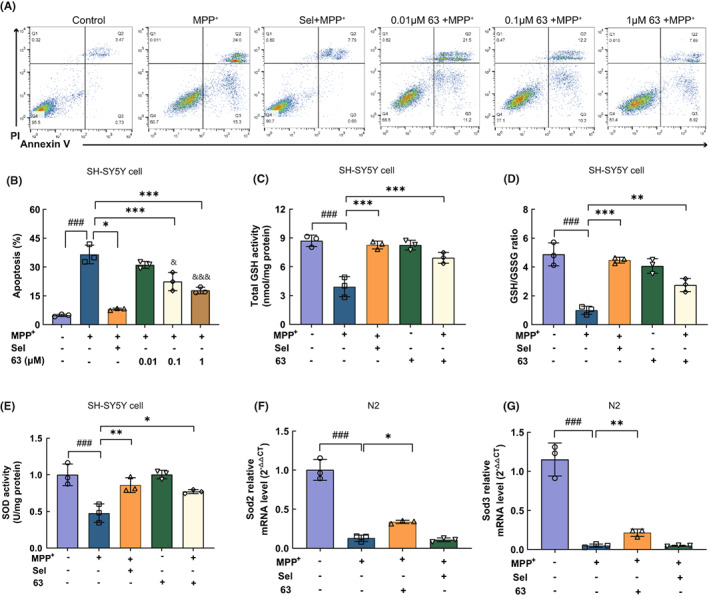
Amelioration of compound 63 on MPP^+^‐induced apoptosis and oxidative stress in SH‐SY5Y cells and N2 *C. elegans*. (A, B) Flow cytometric analyses of Annexin V‐PI staining of apoptotic SH‐SY5Y cells after the treatment of compounds 63. (C–E) Effects of compound 63 on the MPP^+^‐induced oxidative stress of SH‐SY5Y cells were examined by the levels of GSH, SOD, and the ratio of GSH/GSSG ratio. SH‐SY5Y cells were treated with compound 63 and selegiline at 1 μM. (F, G) Effects of compound 63 on the MPP^+^‐induced oxidative stress of N2 *C. elegans* were detected by *Sod2* and *Sod3* mRNA levels. N2 *C. elegans* were treated with compound 63 and selegiline at 5 μM. Differences between the treatment groups were assessed using one‐way anovas, followed by Dunnett's multiple comparisons test or Šídák's multiple comparisons test. Data were expressed as mean ± SEM, ^###^ *p* < 0.001 compared with the control group; *p < 0.05, **p < 0.01, and ***p < 0.001 compared with the MPP^+^ group; ^&^ *p* < 0.05 and ^&&&^ *p* < 0.001 compared with the 0.01 μM‐ compound 63 treatment group. All experiments were performed in triplicate.

The effect of different concentrations of compound 63 on the viability of SH‐SY5Y cells and primary neurons was detected (Figure S2A,E). Furthermore, three concentrations of compound 63 (0.01, 0.1, and 1 μM) were chosen to investigate the effect on apoptosis of MPP^+^‐induced SH‐SY5Y cells and the selegiline (1 μM) was selected as a positive control.^36^ The apoptosis of SH‐SY5Y cells was enhanced by MPP^+^ exposure which was notably restored by compound 63 in a dose‐dependent manner (Figure 6A,B). Additionally, significantly increased levels of GSH, SOD and the ratio of GSH/GSSG were detected in the compound 63‐treated group, which declined in MPP^+^‐induced SH‐SY5Y cells (Figure 6C–E). In vivo, the mRNA levels of *Sod2* and *Sod3* were reversed by compound 63 treatment in MPP^+^‐induced oxidative stress in the N2 *C. elegans* model (Figure 6F,G). These results suggested that compound 63 could increase cell viability, inhibit apoptosis, and reduce oxidative stress induced by MPP^+^ in SH‐SY5Y cells and *C. elegans* model.

**FIGURE 7:**
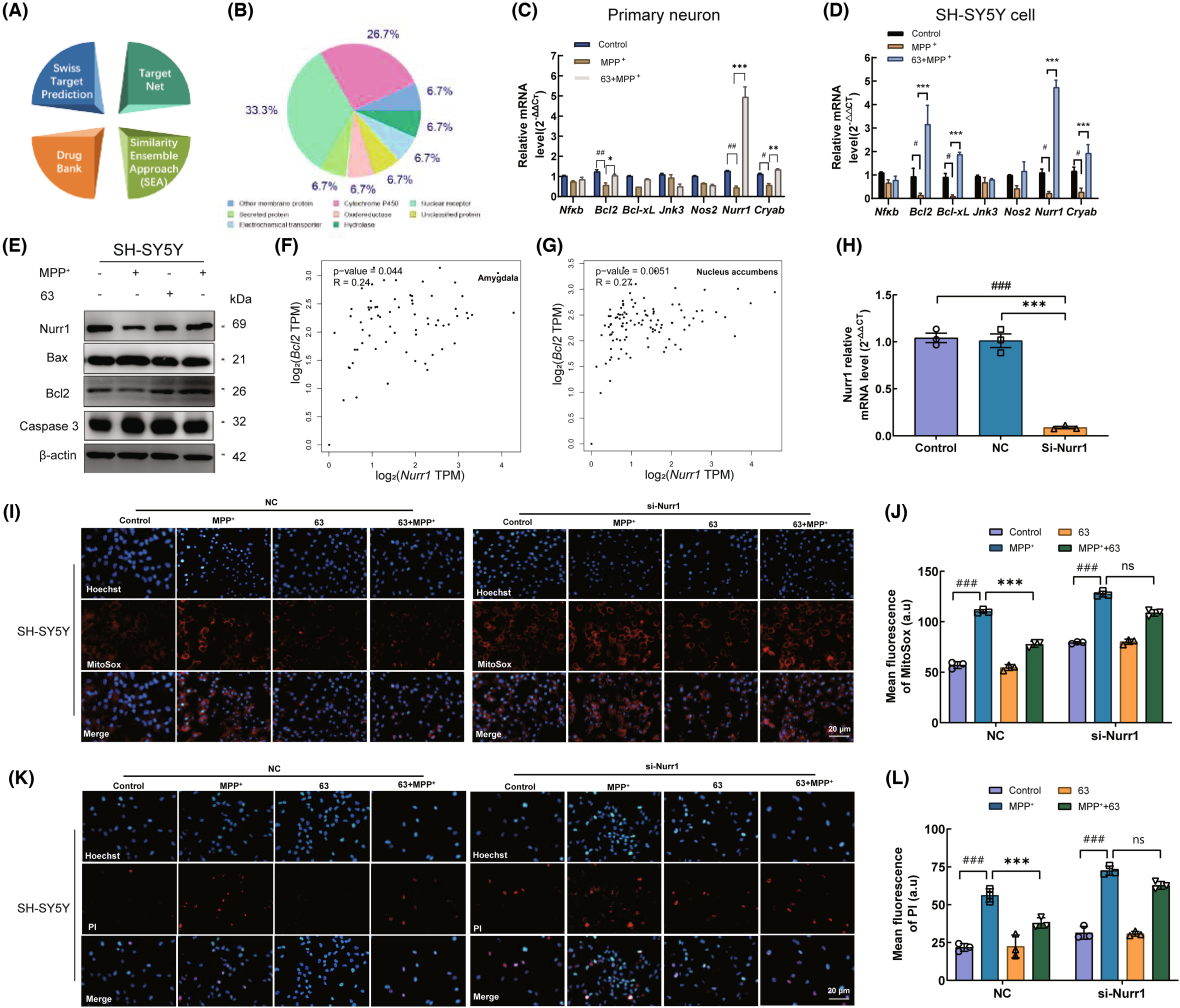
Inhibition of compound 63 on MPP^+^‐induced neuronal apoptosis by Nurr1. (A, B) Target prediction database and possible target analysis of compound 63. (C, D) Levels of *Nfκb*, *Bcl2*, *Bcl‐x*, *Jnk3*, *Nos2*, *Nurr1*, and *Cryab* mRNA expression in primary neurons (C) and SH‐SY5Y cells (D). (E) Western blot analysis of Nurr1, Bcl2, Bax, and Caspase3 expression in SH‐SY5Y cells. (F, G) Correlation analysis of Nurr1 and Bcl2 in the amygdala and nucleus accumbens. (H) The efficiency of Nurr1 knockdown after the transfection of siRNA by QPCR. (I, K) Representative immunofluorescence images of mitoSox (I) and PI (K) in SH‐SY5Y cells. (J, L) Mean fluorescence intensity analysis of MitoSox and PI in SH‐SY5Y cells. Nuclei were stained with Hoechst (blue). Differences between the treatment groups were assessed using one‐way anovas, followed by Dunnett's multiple comparisons test or Šídák's multiple comparisons test. Data were expressed as mean ± SEM, ^#^ *p* < 0.05, ^##^ *p* < 0.01, and ^###^ *p* < 0.001 compared with the control group; *p < 0.05, **p < 0.01 and ***p < 0.001 compared with the MPP^+^ group. All experiments were performed in triplicate.

Furthermore, Target Net, Swiss Target Prediction, Drug Bank, and Similarity Ensemble Approach databases were used to predict potential targets of compound 63. Interestingly, predicted results indicated that nuclear receptors might be the key element for the efficacy of compound 63, which accounted for 33% of all targets (Figure 7A,B). Based on potential targets from four databases, *NfκB*, *Bcl2*, *Bcl‐xl*, *Nos2*, *Nurr1*, and *Cryab* mRNA in SH‐SY5Y cells and primary neurons were further detected. Q‐PCR results in both SH‐SY5Y cells and mouse primary neurons suggested that Nurr1 was notably upregulated by compound 63 (Figure 7C,D). Meanwhile, compound 63 could markedly upregulate the expression of anti‐apoptotic protein Bcl2 and remarkably downregulate the level of Bax and Caspase3 (Figure S4 and Figure S3A–D). The correlation analysis showed that Nurr1 was positively correlated with Bcl2 in the amygdala and nucleus accumbens (Figure 7F,G), which were closely related to neurodegenerative diseases.^37^, ^38^ While the expression of Nurr1 was suppressed with transfected si‐RNA (Figure 7H and Figure S3E,F), compound 63 failed to exhibit a significant neuroprotection ability by MitoSox and PI fluorescence in SH‐SY5Y cells (Figure 7I–L). The above results implied that compound 63 could perform the neuroprotective effect by upregulating the expression of Nurr1 and inhibiting neuronal apoptosis signaling pathways.

## 4 DISCUSSION

The prevalence of neurodegenerative disorders is increasing, however, there is no effective cure for these diseases up to now. Oxidative stress, protein misfolding and aggregation, neuroinflammation, and programmed cell death have been identified to regulate the initiation and progression of neurodegenerative diseases.^39^, ^40^, ^41^ Besides, numerous studies have proved that mitochondrial dysfunction is an important factor contributing to Parkinson's disease.^42^, ^43^, ^44^ Unfortunately, all neurodegenerative diseases are presently incurable and only palliative treatments are available, which urges us to try our best to develop more effective drugs.

Many marine natural products have a potent inhibition of oxidative stress, apoptosis, and neuroinflammation.^45^, ^46^, ^47^ The biocompatibility of marine natural compounds is higher than that of synthetic drugs. Therefore, greater attention has been drawn to the clinical application of marine natural compounds for neurodegenerative diseases such as PD. Currently, the South China Sea coral‐derived compounds have shown various pharmacological activities, including anti‐inflammatory, anti‐nociceptive, and neuroprotective effects.^48^, ^49^ In this study, 101 compounds derived from deep ocean corals were systematically screened and three compounds (No. 63, 68, and 74) performed superior neuroprotective activity. However, compared with compounds 68 and 74, compound 63 performed a better effect on improving MPP^+^‐induced mitochondrial oxidative damage and apoptosis, which was consistent with the results in the *C. elegans* model. Hence, compound 63 was selected as the lead compound for further consideration.

*Caenorhabditis elegans*, which has most human genes, is the first multicellular organism with a completely sequenced genome and is usually used for drug discovery.^50^, ^51^ As a well‐established model organism, *C. elegans* offers several advantages with easy genetic manipulation, convenient accessibility, and low cost. The incubation of *C. elegans* with MPP^+^ has been applied to evaluate anti‐PD drugs, which can cause dopamine‐dependent behavior defects and the loss of dopamine neurons.^52^, ^53^ Our results showed that compound 63 could notably increase the survival rate of the worms and protect against the MPP^+^‐induced toxicity in DA neurons of the *C. elegans* model.

Nurr1 is a nuclear transcription factor that plays a critical role in both the growth and maturation stages of dopaminergic neurons. In the early stages, Nurr1 is referred to promote the differentiation and neurogenesis of dopaminergic neurons and plays an important role in regulating DA metabolism, neurotransmission, axonal growth, and mitochondrial function.^54^ In addition, multiple stimuli, such as inflammatory signaling, hormones, and calcium, can rapidly induce Nurr1 transcription.^55^, ^56^ The reduction of Nurr1 has been observed in PD patients relative to normal subjects, which significantly increases the risk for disease progress.^57^, ^58^ In this study, the drug‐target interaction prediction of compound 63 indicated that most of the enriched biological targets were nuclear receptors. The mRNA and protein levels of Nurr1 were markedly upregulated by compound 63, suggesting that it might exert neuroprotective effects by activating the expression of Nurr1 and further inhibiting apoptosis‐related pathways.

Collectively, compound 63, screened from 101 marine natural compounds, could markedly ameliorate mitochondrial dysfunction and cell damage in SH‐SY5Y cell models. Furthermore, it could notably increase the survival rate and improve the motility disorder of *C. elegans* under the MPP^+^ exposure, which further confirmed the neuroprotective activity of compound 63. And its potential mechanism might activate the expression of Nurr1 and inhibit neuronal apoptosis signaling pathways, which was worthy of further in‐depth investigation in the future. These findings might provide a novel strategy for drug discovery in the treatment of Parkinson's disease.

## AUTHOR CONTRIBUTIONS

Gang Hu and Yang Liu conceived and designed the study. The natural compounds were provided by Bao Chen and Yue‐Wei Guo. Jian‐Wei Su, Pei Yang, Mei‐Mei Xing, and Xia‐Hong Xie performed the experiments and analyzed the data. Jian‐Wei Su and Pei Yang wrote the manuscript. Jian‐Hua Ding provided technical support. Yang Liu and Ming Lu revised the manuscript. All the authors have read and approved the final version of the manuscript.

## CONFLICT OF INTEREST

All the authors declare no conflict of interest.

## Supporting information


Appendix S1

Figure S4

## Data Availability

The original contributions presented in the study are included in the article/supplementary materials, further inquiries can be directed to the corresponding author.
