# Supplementary material for: Neuroprotective effects of a lead compound from coral via modulation of the orphan nuclear receptor Nurr1
Source: CNS Neurosci Ther. 2022 Nov 23;29(3):893–906. doi: 10.1111/cns.14025 (PMC9928544; doi:10.1111/cns.14025)
Supplement: Supplementary file 1 — Appendix S1 [file CNS-29-893-s002.docx]

**Supplementary Materials**

**Neuroprotective effects of a lead compound from coral via modulation of the orphan nuclear receptor Nurr1**

Jian-Wei Su^1§^, Pei Yang ^1§^, Mei-Mei Xing^1^, Bao Chen2, Xia-Hong Xie^1^, Jianhua Ding^3^, Ming Lu^3^, Yang Liu ^1*^, Yue-Wei Guo^2*^, Gang Hu ^1*^

*^1^ Department of Pharmacology, School of Medicine and Holistic Integrative Medicine, Nanjing University of Chinese Medicine, Nanjing, Jiangsu 210023, China.*

*^2^ State Key Laboratory of Drug Research, Shanghai Institute of Materia Medica, Chinese Academy of Sciences, Shanghai 201203, China.*

*^3^ Jiangsu Key Laboratory of Neurodegeneration, Department of Pharmacology, Nanjing Medical University, Nanjing, 211116, China.*

[§] The authors contribute equally to this work.

***Correspondence**

Gang Hu, M.D., Ph.D

Department of Pharmacology, Nanjing University of Chinese Medicine, 138 Xianlin Avenue, Nanjing, Jiangsu 210023, China.

Email: neuropha@njmu.edu.cn

Yue-Wei Guo, M.D., Ph.D.

State Key Laboratory of Drug Research, Shanghai Institute of Materia Medica, Chinese Academy of Sciences 555 Zu Chong Zhi Road, Zhangjiang Hi-Tech Park, Shanghai, 201203, China

Email: [ywguo@simm.ac.cn](mailto:ywguo@simm.ac.cn)

Yang Liu, M.D., Ph.D

Department of Pharmacology, Nanjing University of Chinese Medicine, 138 Xianlin Avenue, Nanjing, Jiangsu 210023, China.

Email: [liuyang@njmu.edu.cn](mailto:liuyang@njmu.edu.cn)


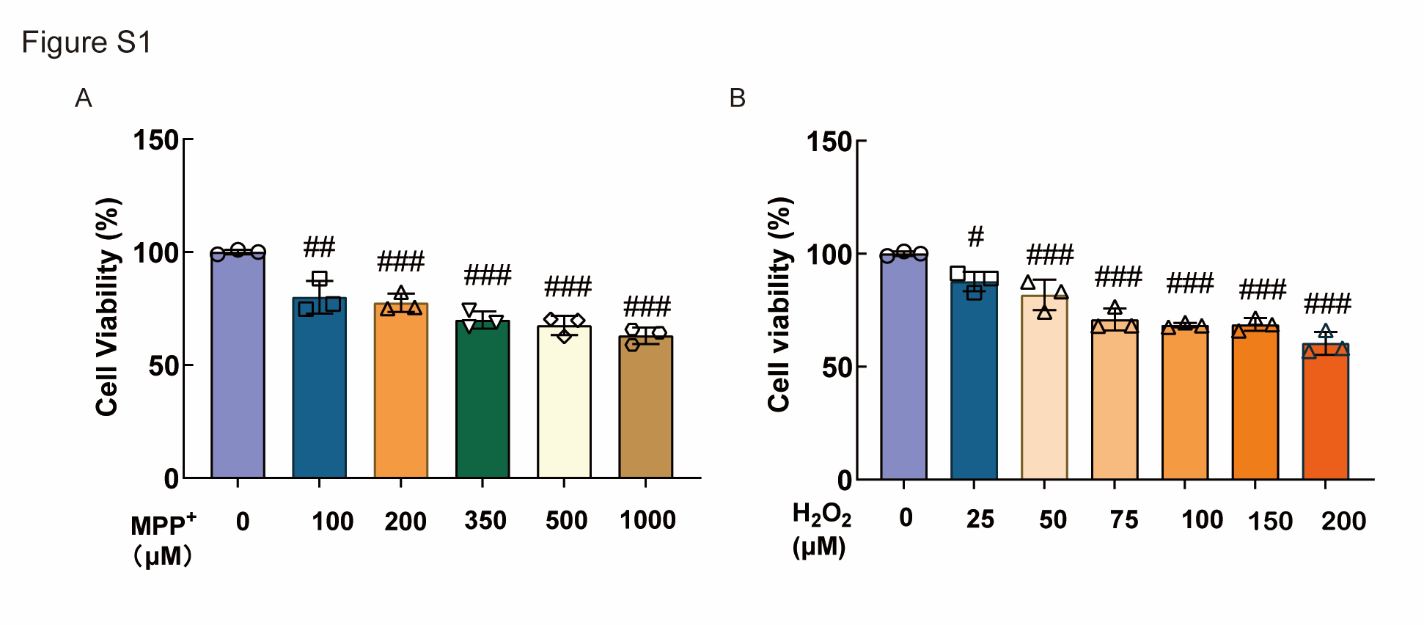


**Figure S1 Effects of the different concentrations of MPP^+^/H_2_O_2_ on the viability of SH-SY5Y cells.** (A) The cell viability of MPP^+^-induced SH-SY5Y cells. (B) The cell viability of H_2_O_2_-induced SH-SY5Y cells. Differences between the treatment groups were assessed using one-way ANOVAs, followed by Dunnett's multiple comparisons test. Data were expressed as mean ± SEM, ^#^*p* < 0.05, ^##^*p* < 0.01, and ^###^*p* < 0.001 compared with the control group. All experiments were performed in triplicate.


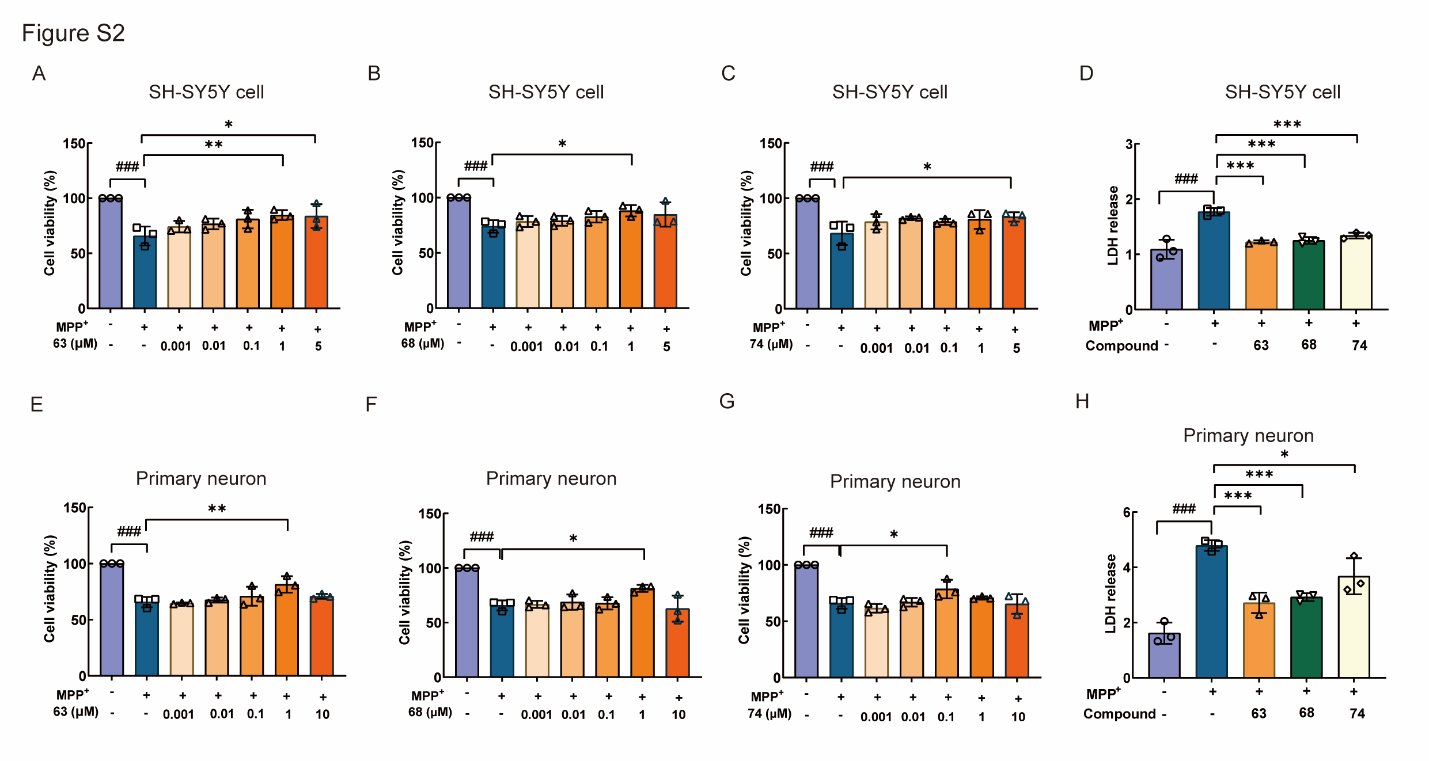


**Figure S2 Neuroprotective activities of compounds 63, 68, and 74 on MPP^+^-induced SH-SY5Y cells and primary neurons.** (A-C) The effect of compounds 63 (A), 68 (B) and 74 (C) on the viability of SH-SY5Y cells. (D) LDH release of SH-SY5Y cells after the treatment of compounds 63, 68, or 74. (E-G) The effect of compounds 63 (E), 68 (F) and 74 (G) on the viability of primary neurons. (H) LDH release of primary neurons after the treatment of compounds 63, 68, or 74. Differences between the treatment groups were assessed using one-way ANOVAs, followed by Šídák's multiple comparisons test or Dunnett's multiple comparisons test. Data were expressed as mean ± SEM, ^###^*p* < 0.001 compared with the control group; ^*^*p* < 0.05, ^**^*p* < 0.01 and ^***^*p* < 0.001 compared with the MPP^+^ group. All experiments were performed in triplicate.


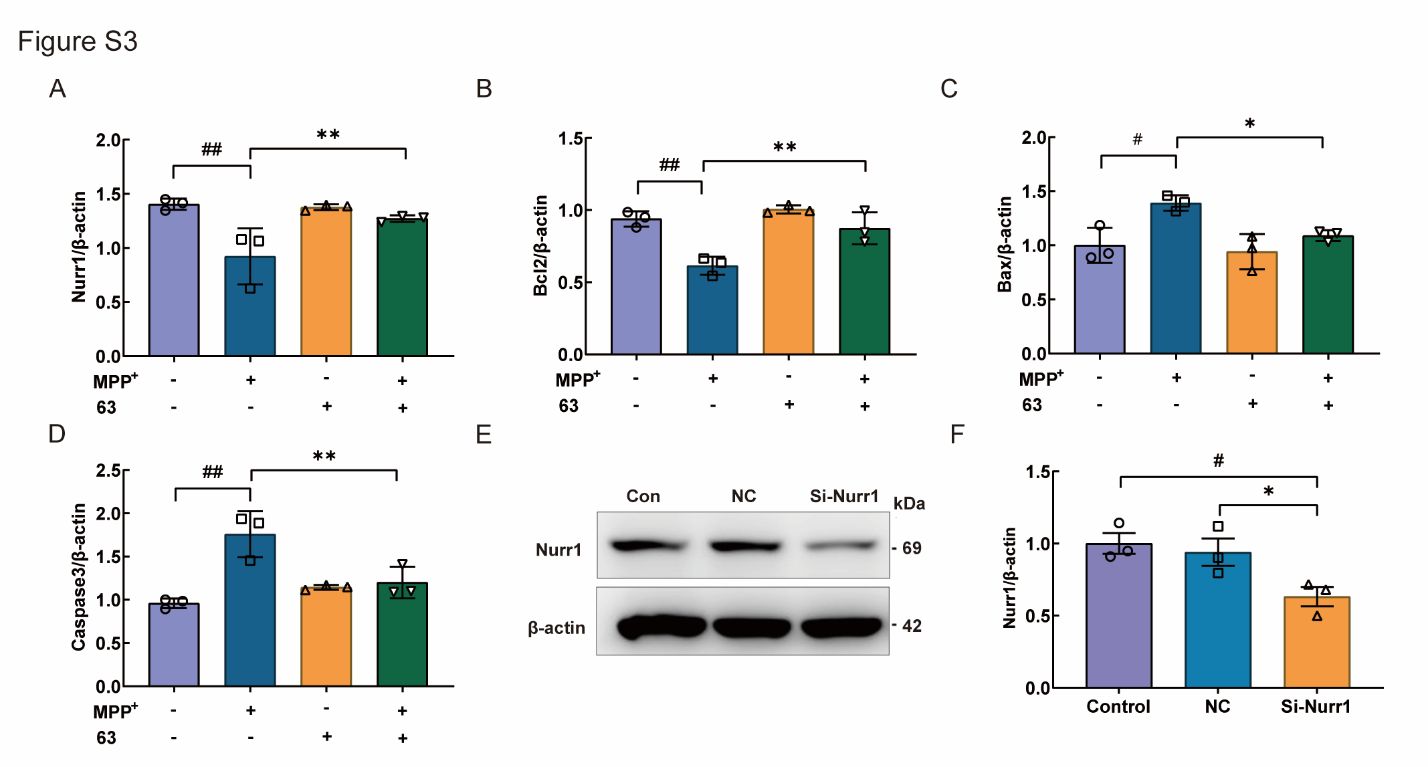


**Figure S3 The protein expression of Nurr1, Bcl2, Bax, Caspase-3, and transfection efficacy of si-Nurr1 in SH-SY5Y cells.**

(A-D) Effects of compound 63 on expression levels of Nurr1 (A), Bcl2 (B), Bax (C), and Caspase-3 (D) proteins. ^#^*p* < 0.05 and ^##^*p* < 0.01 compared to the control group; ^*^*p* < 0.05, ^**^*p* < 0.01 compared with the MPP^+^ group. (E) Nurr1 protein expression in SH-SY5Y cells after the transfection with Nurr1 siRNA or negative control by western blotting. (F) Densitometric analyses of Nurr1versus β-actin; ^#^*p* < 0.05 compared to the control group; ^*^*p* < 0.05 compared the Nurr1 siRNA group to the negative control group; Differences between the treatment groups were assessed using one-way ANOVAs, followed by Šídák's multiple comparisons test or Dunnett's multiple comparisons test. Data were expressed as mean ± SEM. All experiments were performed in triplicate.

**Table S1 The sequences of the primers for RT-qPCR**

| Name | Forward | Reverse |
| --- | --- | --- |
| *m-Bcl2* | CCTGTGGATGACTGAGTACCTG | AGCCAGGAGAAATCAAACAGAGG |
| *m-Bclx* | GCCACCTATCTGAATGACCACC | AGGAACCAGCGGTTGAAGCGC |
| *m-Jnk3* | CGCTACCAGAACCTGAAGCCAA | GGCGTGAGTTTGGTTCTGGAAG |
| *m-Nos2* | GAGACAGGGAAGTCTGAAGCAC | CCAGCAGTAGTTGCTCCTCTTC |
| *m-Nurr1* | CCGCCGAAATCGTTGTCAGTAC | TTCGGCTTCGAGGGTAAACGAC |
| *m-Nfκb* | GCTGCCAAAGAAGGACACGACA | GGCAGGCTATTGCTCATCACAG |
| *m-Cryab* | CGGAGGAACTCAAAGTCAAGGTT | ATGGTGAGAGGATCCACATCGG |
| *m-Gapdh* | AACGACCCCTTCATTGAC | TCCACGACATACTCAGCAC |
| *h-Bcl2* | ATCGCCCTGTGGATGACTGAGT | GCCAGGAGAAATCAAACAGAGGC |
| *h-Bclx* | GCCACTTACCTGAATGACCACC | AACCAGCGGTTGAAGCGTTCCT |
| *h-Jnk3* | TATGTGGAGAATCGGCCCAAG | GCTTTGAGTTTATTGTGCTCGG |
| *h-Nos2* | GCCTCGCTCTGGAAAGA | TCCATGCAGACAACCTT |
| *h-Nurr1* | AAACTGCCCAGTGGACAAGCGT | GCTCTTCGGTTTCGAGGGCAAA |
| *h-Nfκb* | GCAGCACTACTTCTTGACCACC | TCTGCTCCTGAGCATTGACGTC |
| *h-Cryab* | ACTTCCCTGAGTCCCTTCTACC | GGAGAAGTGCTTCACATCCAGG |
| *h-Gapdh* | GCACCGTCAAGGCTGAGAAC | TGGTGAAGACGCCAGTGGA |
| *c-Sod2* | ACCATCGGCGGAGTTGCTCA | AGCGTGCTCCCAGACGTCAA |
| *c-Sod3* | GTGGTGGACACATCAATC | AAGTGGGACCATTCCTTC |
| *c-Actin* | CCATCATGAAGTGCGACATTG | CATGGTTGATGGGGCAAGAG |

*Note: m-mouse; h-human;c-C. elegans*

**Table S2 The effect of different compounds on the viability of SH-SY5Y cells.**

| Number | Mean ± SEM | Number | Mean ± SEM | Number | Mean ± SEM |
| --- | --- | --- | --- | --- | --- |
| 1 | 98.33 ± 5.03 | 41 | 91.50 ± 8.05 | 81 | 99.67 ± 2.08 |
| 2 | 82.83 ± 8.28 | 42 | 83.00 ± 5.29 | 82 | 104.33 ± 7.51 |
| 3 | 77.67 ± 2.02 | 43 | 84.33 ± 8.02 | 83 | 85.47 ± 2.66 |
| 4 | 87.67 ± 5.51 | 44 | 84.33 ± 9.50 | 84 | 94.00 ± 5.20 |
| 5 | 94.13 ± 3.20 | 45 | 89.63 ± 5.45 | 85 | 84.33 ± 6.66 |
| 6 | 91.97 ± 1.77 | 46 | 78.70 ± 8.87 | 86 | 90.00 ± 5.00 |
| 7 | 84.80 ± 5.07 | 47 | 76.60 ± 5.91 | 87 | 81.00 ± 3.61 |
| 8 | 85.47 ± 7.34 | 48 | 85.17 ± 7.74 | 88 | 86.43 ± 5.88 |
| 9 | 76.23 ± 8.17 | 49 | 87.67 ± 6.35 | 89 | 87.70 ± 1.54 |
| 10 | 77.27 ± 7.57 | 50 | 86.67 ± 1.15 | 90 | 84.00 ± 3.00 |
| 11 | 92.67 ± 9.52 | 51 | 77.00 ± 4.00 | 91 | 93.67 ± 7.77 |
| 12 | 87.00 ± 9.54 | 52 | 97.90±7.45 | 92 | 81.20 ± 2.50 |
| 13 | 88.50 ± 8.76 | 53 | 95.60 ± 3.95 | 93 | 90.73 ± 6.51 |
| 14 | 102.40 ± 5.33 | 54 | 85.70 ± 8.07 | 94 | 70.77 ± 5.04 |
| 15 | 87.40 ± 8.31 | 55 | 92.73 ± 5.53 | 95 | 68.00 ± 3.00 |
| 16 | 86.70 ± 9.01 | 56 | 88.60 ± 2.26 | 96 | 80.03 ± 1.00 |
| 17 | 73.90 ± 6.62 | 57 | 106.40 ± 5.05 | 97 | 81.77 ± 6.37 |
| 18 | 77.37 ± 5.95 | 58 | 103.33 ± 9.45 | 98 | 80.67 ± 4.04 |
| 19 | 97.03 ± 7.75 | 59 | 95.57 ± 5.68 | 99 | 84.00 ± 1.73 |
| 20 | 84.30 ± 3.76 | 60 | 95.67 ± 6.51 | 100 | 88.00 ± 2.65 |
| 21 | 89.07 ± 5.08 | 61 | 91.97 ± 6.29 | 101 | 86.3 3 ± 1.53 |
| 22 | 82.36 ± 7.83 | 62 | 99.00 ± 2.65 |  |  |
| 23 | 83.50 ± 5.22 | 63 | 103.33 ± 3.06 |  |  |
| 24 | 64.63 ± 5.84 | 64 | 95.33 ± 3.21 |  |  |
| 25 | 81.04 ± 7.95 | 65 | 79.60 ± 2.25 |  |  |
| 26 | 57.03 ± 4.72 | 66 | 96.33 ± 9.02 |  |  |
| 27 | 54.27 ± 5.41 | 67 | 96.67 ± 6.35 |  |  |
| 28 | 80.33 ± 7.51 | 68 | 102.67 ± 2.08 |  |  |
| 29 | 82.17 ± 5.75 | 69 | 86.33 ± 5.86 |  |  |
| 30 | 78.00 ± 6.08 | 70 | 84.03 ± 3.95 |  |  |
| 31 | 88.77 ± 4.52 | 71 | 97.00 ± 7.55 |  |  |
| 32 | 84.83 ± 8.28 | 72 | 93.67 ± 2.52 |  |  |
| 33 | 80.83 ± 6.90 | 73 | 102.33 ± 3.06 |  |  |
| 34 | 87.67 ± 8.08 | 74 | 103.00±7.00 |  |  |
| 35 | 90.70 ± 3.75 | 75 | 101.33 ± 4.62 |  |  |
| 36 | 76.77 ± 5.26 | 76 | 89.27 ± 5.51 |  |  |
| 37 | 95.33 ± 3.71 | 77 | 103.67 ± 6.51 |  |  |
| 38 | 96.00 ± 6.16 | 78 | 100.67 ± 8.08 |  |  |
| 39 | 103.43 ± 6.76 | 79 | 89.47 ± 7.39 |  |  |
| 40 | 98.63 ± 5.54 | 80 | 86.40 ± 4.73 |  |  |

Note: SH-SY5Y cells were treated with different compounds at 1 μM. All experiments were performed in triplicate.
