# Supplementary material for: Neuroprotective effects of a lead compound from coral via modulation of the orphan nuclear receptor Nurr1
Source: CNS Neurosci Ther. 2022 Nov 23;29(3):893–906. doi: 10.1111/cns.14025 (PMC9928544; doi:10.1111/cns.14025)

Neuroprotective effects of a lead compound from coral via modulation of the orphan nuclear receptor Nurr1

2022.10.20

Figure 7E

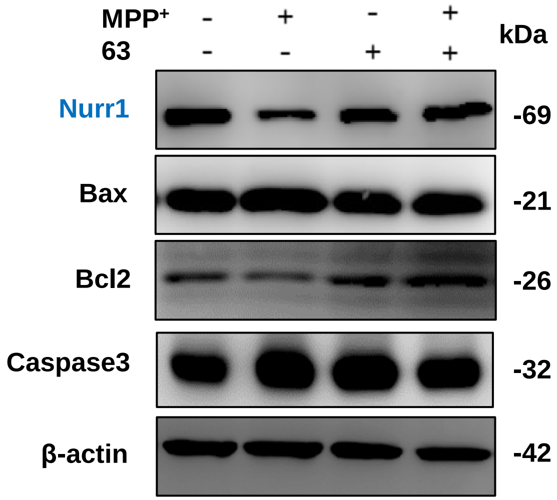

Nurr1

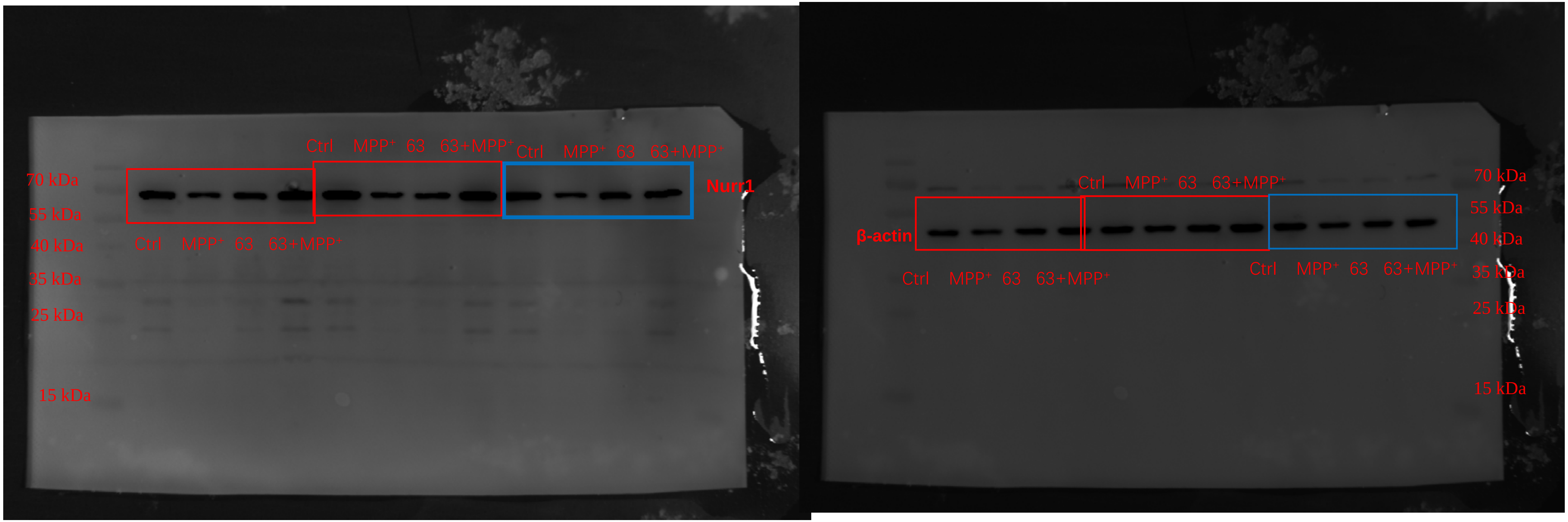

Figure 7E

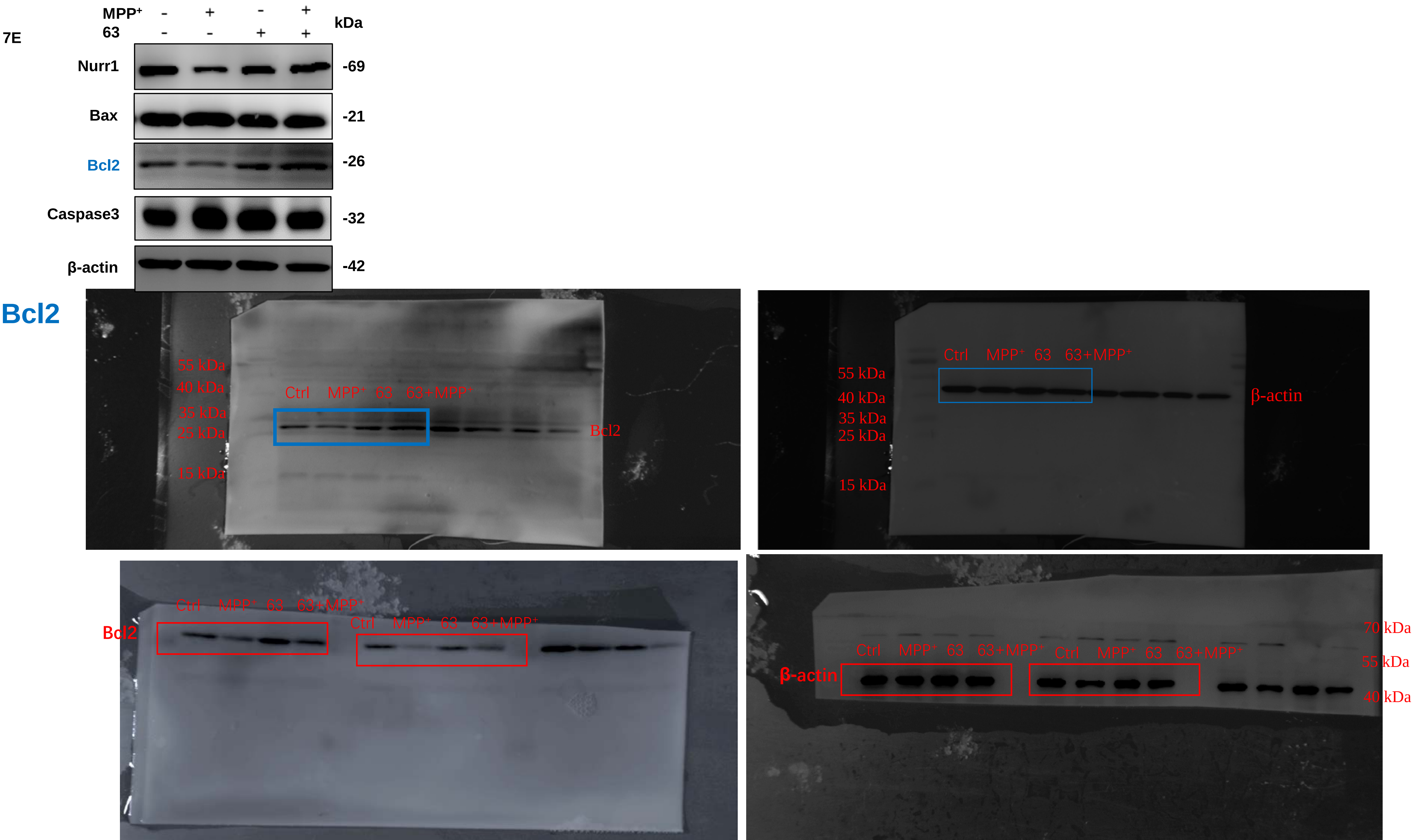

MPP+ - + - +
63 - - + +
kDa
Nurr1 -69
Bax -21
Bcl2 -26
Caspase3 -32
β-actin -42
Bcl2
55 kDa
40 kDa
35 kDa
25 kDa
15 kDa
Ctrl MPP+ 63 63+MPP+
Bcl2
Ctrl MPP+ 63 63+MPP+
β-actin
Bcl2
Ctrl MPP+ 63 63+MPP+
Ctrl MPP+ 63 63+MPP+
β-actin
70 kDa
55 kDa
40 kDa

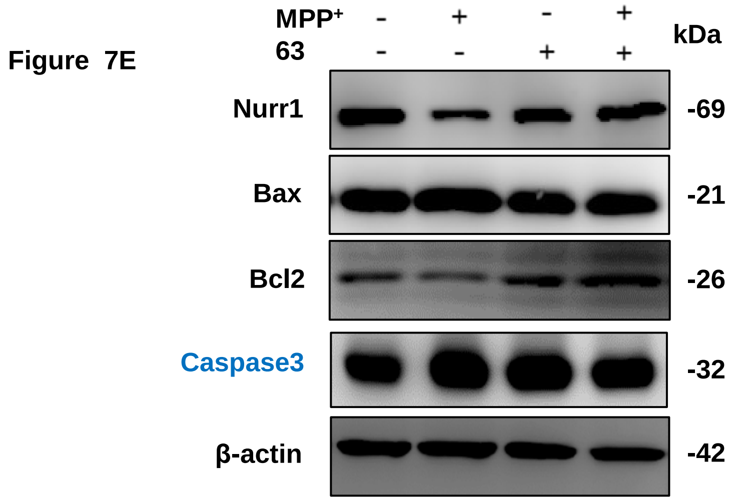
Figure 7E
MPP+ - + - +
63 - - + +
kDa
Nurr1 -69
Bax -21
Bcl2 -26
Caspase3 -32
β-actin -42

Caspase3

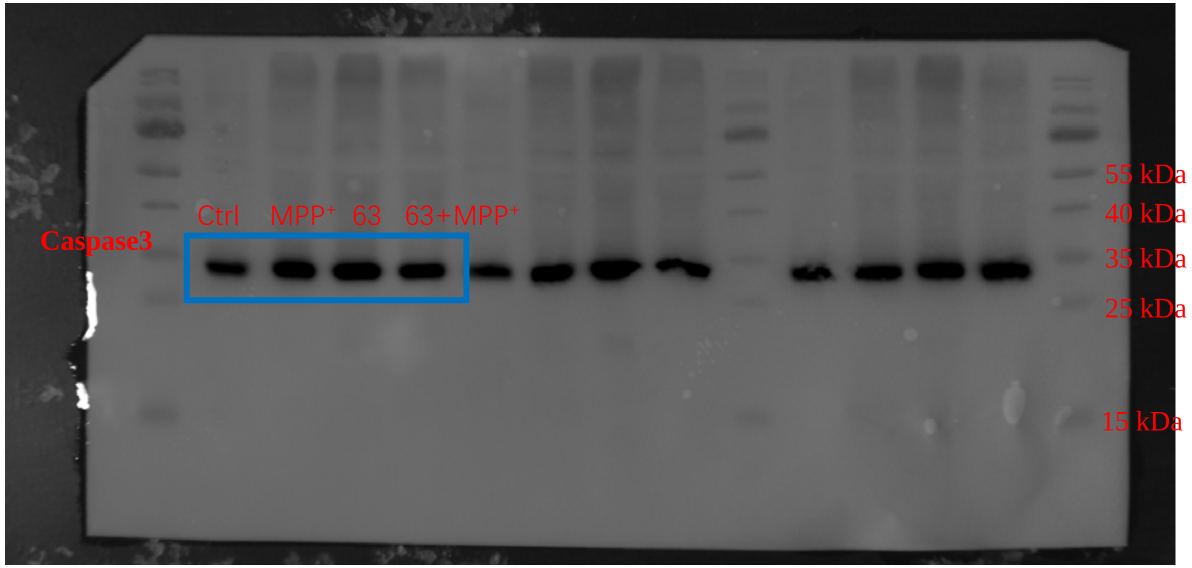
Ctrl MPP+ 63 63+MPP+
Caspase3
55 kDa
40 kDa
35 kDa
25 kDa
15 kDa

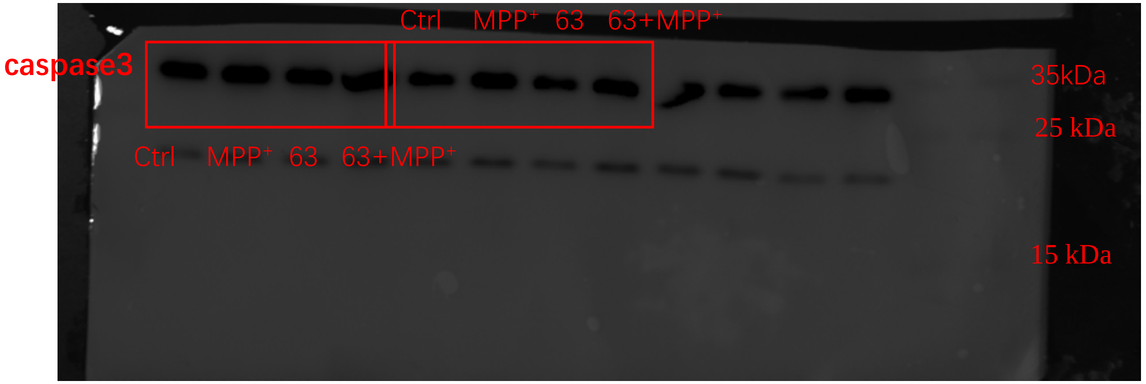
Ctrl MPP+ 63 63+MPP+
caspase3
35kDa
25 kDa
Ctrl MPP+ 63 63+MPP+
15 kDa

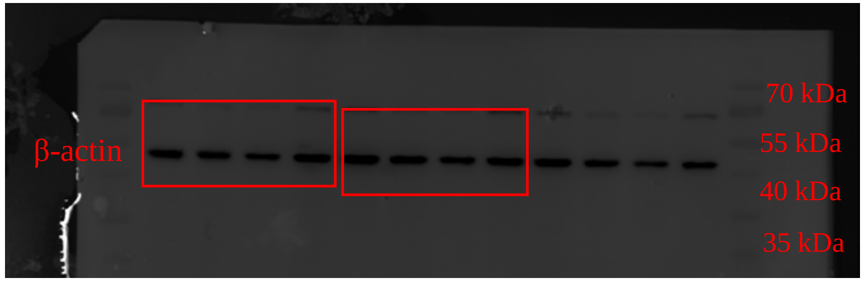
β-actin
70 kDa
55 kDa
40 kDa
35 kDa

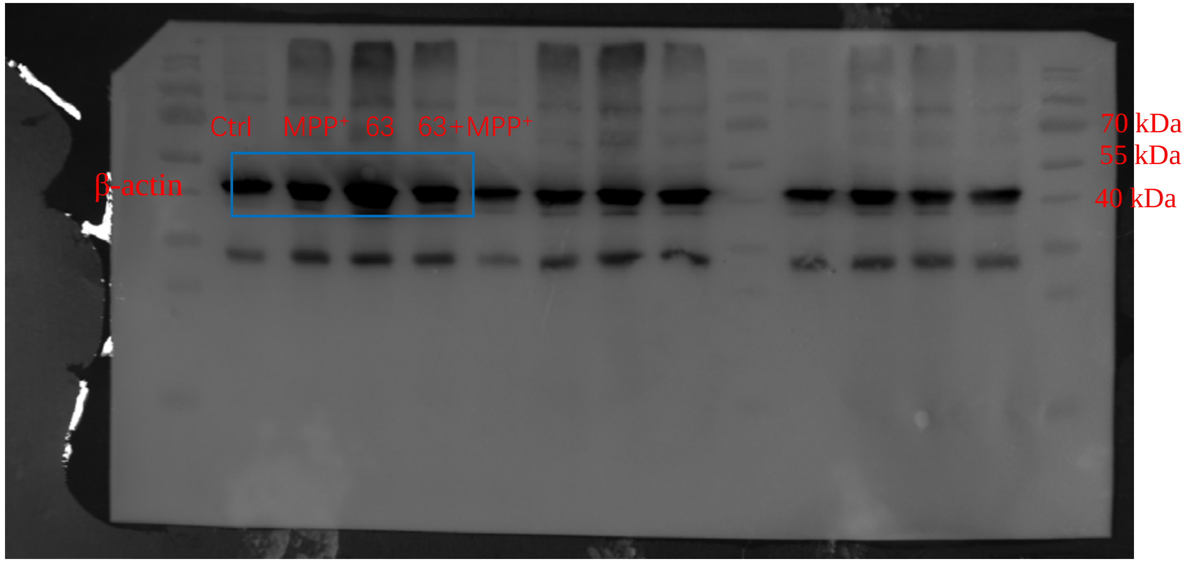
Ctrl MPP+ 63 63+MPP+
β-actin
70 kDa
55 kDa
40 kDa

Figure 7E

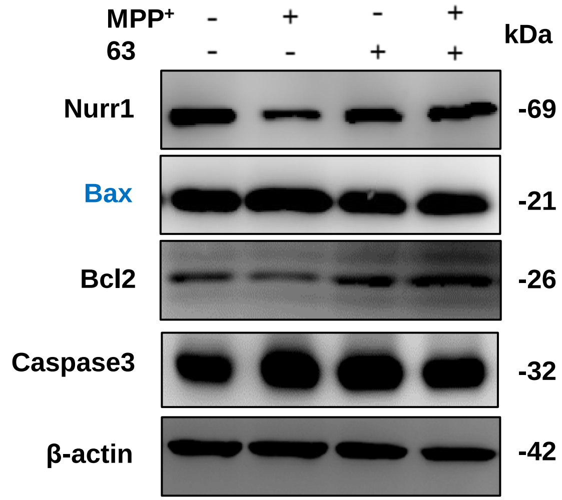

Bax

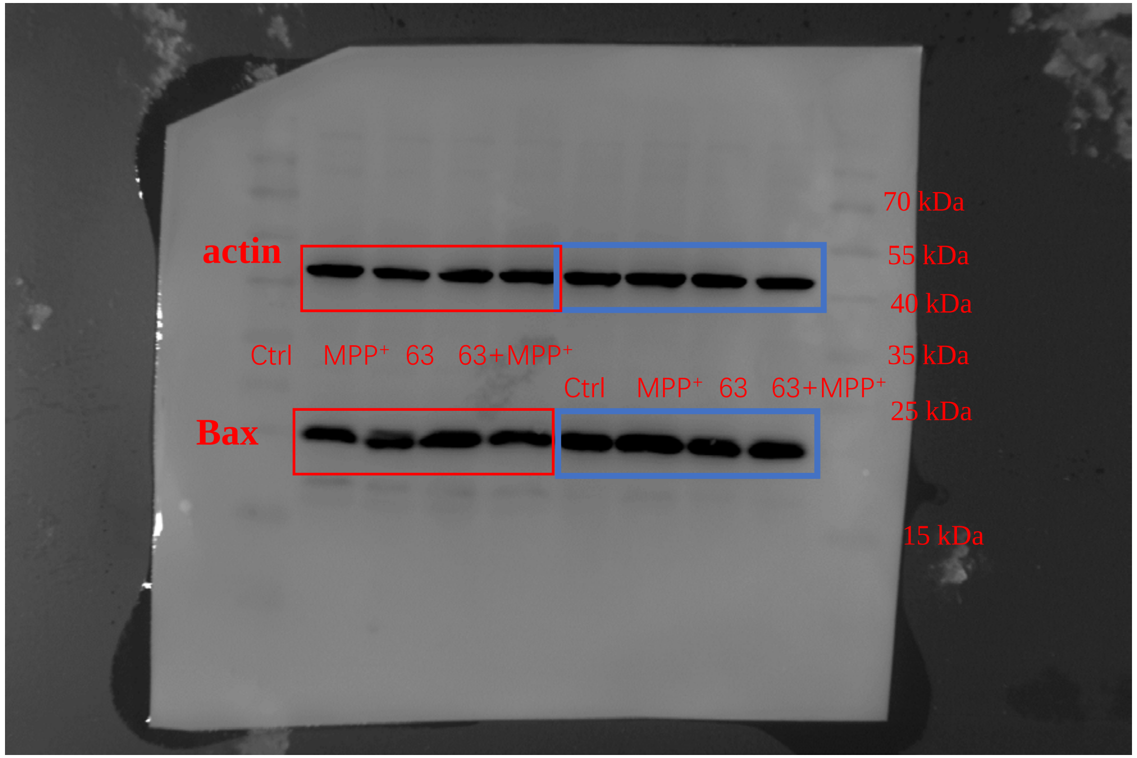

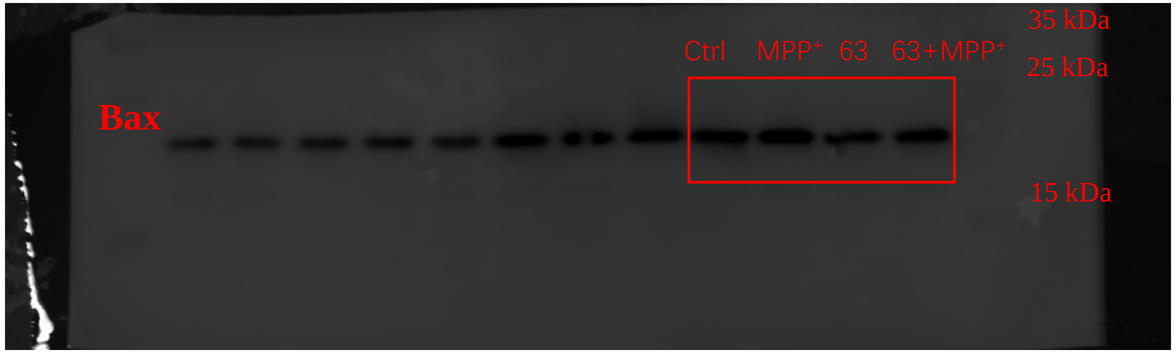

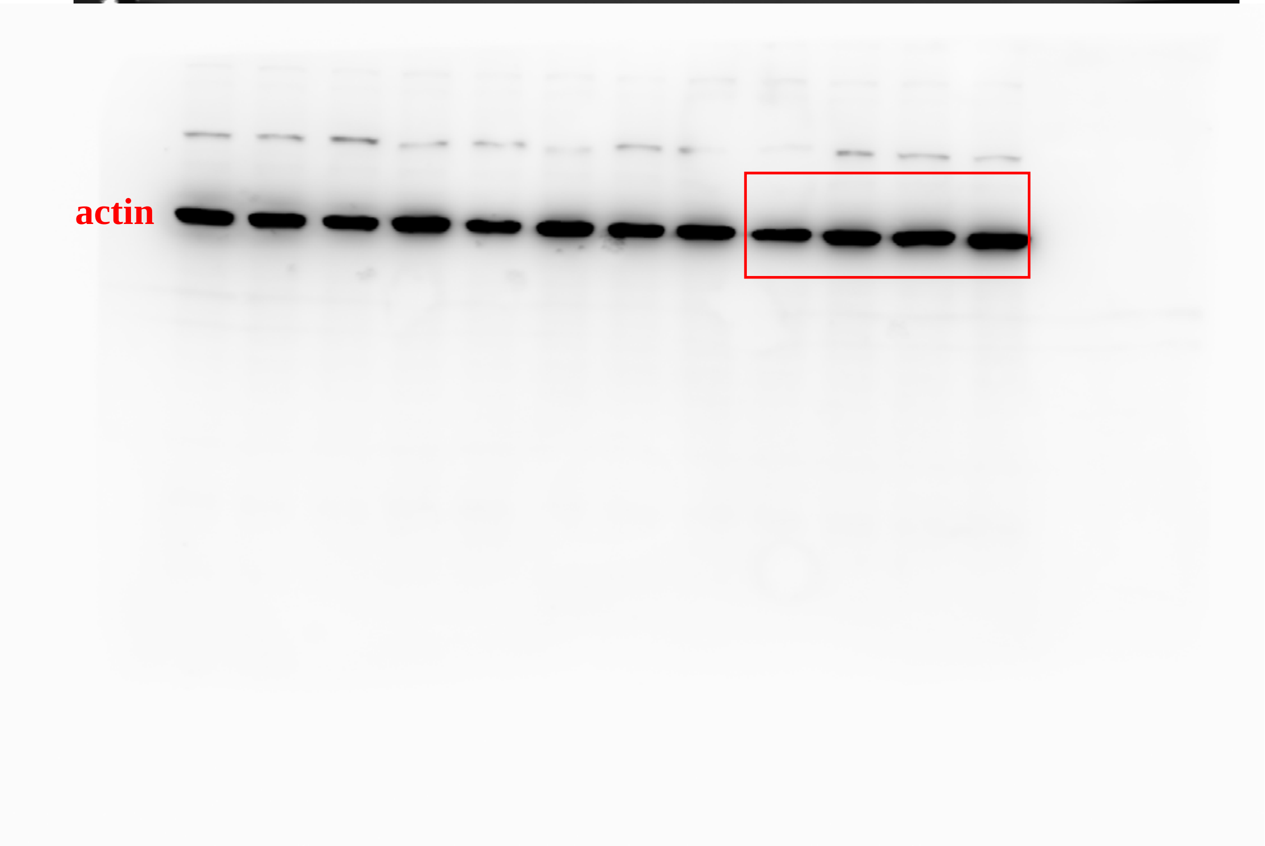

Supplement: Supplementary file 2 — Figure S4 [file CNS-29-893-s001.pdf]
